# A do-it-yourself benchtop device for highly scalable flow synthesis of protein-based nanoparticles

**DOI:** 10.1016/j.ohx.2024.e00554

**Published:** 2024-07-01

**Authors:** Egor S. Korenkov, Vladimir R. Cherkasov, Maxim P. Nikitin

**Affiliations:** a Moscow Center for Advanced Studies, 123592 Moscow, Russia; b Moscow Institute of Physics and Technology (State University), 117303 Moscow, Russia; c Sirius University of Science and Technology, 354340 Sochi, Russia

**Keywords:** Microfluidics, Millifluidics, Nanoparticle synthesis, Protein nanoparticles, Drug delivery, Swift thermal formation

## Abstract

Synthesis of nanoparticles is typically carried out in batch procedures, which offer limited control of parameters, and a narrow range of possible batch volumes. In contrast, flow synthesis systems, usually having a microfluidic chip as a crucial part, are devoid of these drawbacks. However, large scale devices – millifluidic systems – may offer several advantages over microfluidic systems, such as easier and cheaper production, enhanced throughput, and reduced channel clogging. Here we report a millifluidic system for the generation of protein nanoparticles, using the flow format of the original swift thermal formation technology (STF), which can process batch volume ranging from 100 µl to any practically significant amount. Capabilities of the system are demonstrated with model synthesis of Epirubicin-encapsulated BSA nanoparticles. A better degree of scalability of the synthesis over batch procedure is shown: with a 10-fold working volume increase, hydrodynamic diameter and loading capacity changed by only 10 % and 1 % respectively, compared to 60 % and 30 % for the batch synthesis. Additionally, we provide all engineering drawings, electrical circuits, programming code and nuances of assembly and operation, so that our findings can be easily reproduced. The ease of construction of the device and the superior characteristics of the resulting nanoparticles compared to the batch method indicate application potential in both the biomedical research and industrial spheres.

## Graphical abstract

**Figure:**
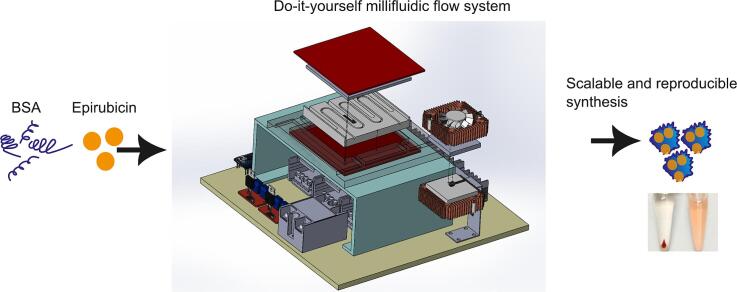

Specifications table

**Table:** 

Hardware name	*Flow reactor for protein nanoparticles synthesis*
Subject area	• Nanomaterials Engineering and materials science
Hardware type	• Biological sample preparation Mechanical engineering and materials science
Open Source License	*CC-BY 4.0*
Cost of Hardware	*$350*
Source File Repository	https://doi.org/10.17632/bptpfpgf5h.2

## 1 Hardware in context

Nanoparticles are widely used in the area of biomedicine, including applications in therapy [1], [2], diagnostics and biosensing [3], [4], bioimaging [5] etc. Among them, use of nanoparticles as drug carriers is a promising approach to improve performance of therapeutic substances by increasing their solubility, bioavailability, reducing toxicity, etc. [6].

Protein nanoparticles attract particular attention because of their inherent biocompatibility, biodegradability and non-toxicity [7], [8], [9]. At present, there are clinically approved formulations, such as nanoparticle-bound albumin paclitaxel (abraxane) [10], [11]. Numerous methods for protein nanoparticle synthesis have been reported, typically done in batch manner [12]. Among them, a method of protein nanoparticle synthesis was developed, swift thermal formation (STF) [13]. It was shown that intense but rapid heating can promote protein nanoparticle formation, while minimizing impact on protein function and structure. Comparison of STF to other synthesis methods is summarized in Table S1.

However, for those batch methods, it remains challenging to scale up nanomaterial synthesis while maintaining control over their physicochemical parameters, which significantly hampers lab to clinic/industry transition [14]. Hence, we sought for a possibility to implement STF in a continuous flow manner, as it generally offers much better scalability and parameters control [15]. Usually, such flow systems have a microfluidic chip as their crucial part [16], [17], [18], [19]. Most commonly, the processes rely on variation of coacervation or desolvation methods, pursuing the fast and effective mixing of protein with some kind of antisolvent or trigger. The mixing is assisted by various physical phenomena, such as ultrasonication [18], AC field [17], internal vortices inside oil droplets [20], chip geometry [21], [22].

However, our goal is to construct our setup without resorting to microfluidic chips. This is due to the microscopic nature of the channels, which makes their production a complex and costly process. Additionally, their use may be associated with limited throughputs and an increased risk of channel clogging [23].

The same principles, that are used for microfluidic systems, can be partially transferred to the case of millifluidics, which are flow systems with characteristic dimensions of 1 mm. Millifluidic systems are successfully applied for the synthesis of various nanoparticles types, such colloidal gold [24], [25], [26], quantum dots [27], [28], [29], liposomes [30], [31], polymer nanoparticles [32], [33], [34]. However, to our knowledge, reports on millifluidic synthesis of protein nanoparticles are scarce [35], [36]. In the work by Chen et al. [35] nanoparticles of bovine serum albumin (BSA) are synthesized by millifluidic mixing BSA with crosslinking initiator, which allows to yield small nanoparticles (<20 nm) and additionally load them with another protein. In the work of Seaberg et al. [36] BSA is millifluidically mixed with a copolymer of poly(ethylene glycol) and poly(L-lysine), and treated by ultrasound to synthesize mixed polymer-protein nanoparticles.

To combine the advantages of STF and benefits of a flow synthesis, we developed a millifluidic approach for synthesis of loaded protein nanoparticles for biomedical applications. To demonstrate the capabilities of the system, we synthesized BSA nanoparticles encapsulated with Epirubicin, as an example of a cancer drug delivery system [37]. A key feature of the system is simplicity in switching between optimization scale synthesis (e.g., 0.1 mL) and near industrial scale production (e.g., above 1L) without any change in the system. Additionally, the setup is low-cost (below $350) and uses an open soft- and hardware.

## 2 Hardware description

**Fig. 1:**
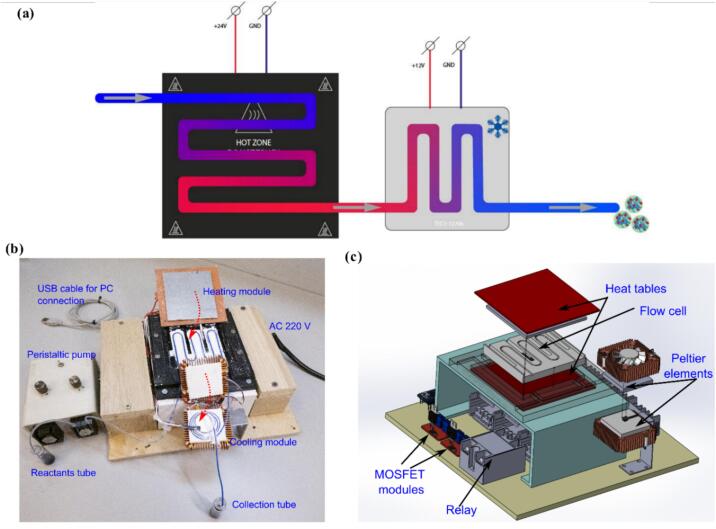
Scheme and setup of the system. (a) Schematic drawing of the flow cell. (b) Photograph of the experimental setup. Upper halves of heating and cooling module during operation are closed (shown by red dotted arrow). For better visibility, tube is filled with 1 g/l methylene blue dye. (c) 3d-model view of the experimental assembly. (For interpretation of the references to color in this figure legend, the reader is referred to the web version of this article).

To develop a scalable synthesis of protein nanoparticles, we constructed a flow heat system. The schematic drawing of the setup is shown in Fig. 1(a), the photograph and 3D-model view are shown in Fig. 1(b, c) respectively. The reaction mixture, in the simplest case consisting of a protein and a moiety to be encapsulated, is pumped with a peristaltic pump through a heating module. There, due to protein heat aggregation, nanoparticles formation takes place. Further, liquid enters the cooling module, which is designed to rapidly stop aggregation and effectively quench the nanoparticles’ formation.

### 2.1 Heating module construction

During the design of the system, special care was taken to ensure that (1) the temperature sensor accurately reported the temperature of the reaction mixture, (2) the temperature distribution throughout the tube was as uniform as possible, and (3) the temperature specified by the user was always achieved and maintained during the synthesis process.

**Fig. 2:**
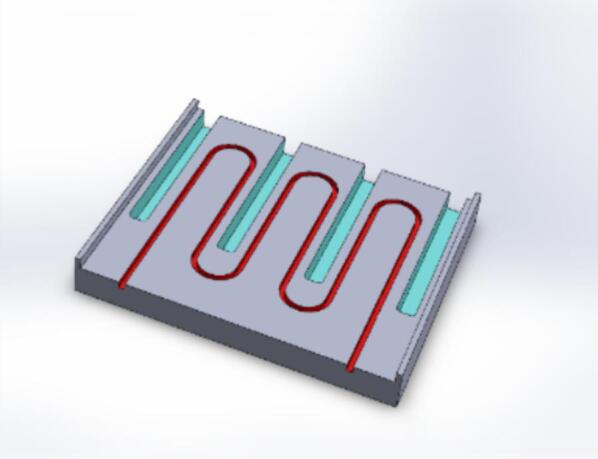
3D-model view of aluminum tube support of the heating module. Tube housing is shown in red, temperature sensors' sockets are shown in blue. (For interpretation of the references to color in this figure legend, the reader is referred to the web version of this article).

To meet the requirement of uniform heat distribution, an aluminum plate with cut-out grooves for temperature sensors and a groove for tubes was designed (Fig. 2). The plate is sandwiched between two heating tables measuring 100x100 mm and a power of 60 W. It serves to evenly redistribute heat between the tube and the sensor. Accurate temperature control is achieved using a proportional-integral-derivative (PID) feedback loop. The DS18B20 temperature sensor sends the difference between the actual and set temperature to the Arduino controller. Based on this value at time t, the controller calculates the output value, output(t):

$$output\left(t\right)=K_{P}∙diff\left(t\right)+K_{I}∙\int_{t-\Delta t}^{t}diff\left(t\right)∙dt+K_{d}∙\;\frac{d}{\mathrm{dt}}diff\left(t\right),$$

where K_P_, K_I_, K_d_ and Δt are selected experimentally. This output value is directly proportional to the power supplied to the heating tables. It is sent as a pulse-width modulation signal to the MOSFET module, which directly regulates the power supplied to the heaters. Values of the coefficients are available in the source code in the repository.

### 2.2 Cooling module construction

**Fig. 3:**
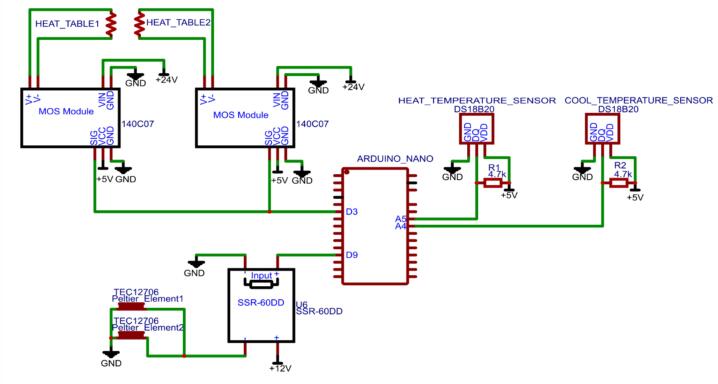
Electrical circuit of the setup.

After the heating module, the tube passes between two 60 W Peltier elements measuring 40x40 mm, which in turn are surrounded by two heat sinks with air fans. The cooling module implements a simpler feedback loop. The relay turns the cooling on and off with a threshold of 5 ˚C. With this control scheme, the actual temperature fluctuates between 2 ˚C and 7 ˚C with a period of approximately 10 s. The electrical circuit implementing this feedback control for the heating and cooling module is shown in Fig. 3.

## 3 Design files summary

### 3.1 Bill of materials summary

Technical drawings and 3D-models are listed in Table 1, and components (or their close analogs) to build the device are listed in Table 2. The heating elements are chosen as the smallest heat tables for 3D-printers. Current limit for MOSFET modules should be above 1A, and for the relay it should be above 20A. Bigger heat sinks for cooling modules can also be used, especially if water cooling is used instead of air cooling. Any peristaltic pump can be used, as long as it can provide a desirable flow rate.

**Table 1:** Design file summary.

Design file name	File type	Open-source license	Location of the file
*Flow heat cell*	*CAD files(.step,.stl)*	*CC-BY 4.0*	*https://doi.org/10.17632/bptpfpgf5h.2*
*Support for heat table*	*CAD files(.step,.stl)*	*CC-BY 4.0*	*https://doi.org/10.17632/bptpfpgf5h.2*
Pi shaped support	*CAD files(.step,.stl)*	*CC-BY 4.0*	*https://doi.org/10.17632/bptpfpgf5h.2*
Final assembly	*CAD files(.step)*	*CC-BY 4.0*	*https://doi.org/10.17632/bptpfpgf5h.2*
Circuit_diagram	*PDF file*	*CC-BY 4.0*	*https://doi.org/10.17632/bptpfpgf5h.2*
Source_code	*Arduino(.ino)*	*CC-BY 4.0*	*https://doi.org/10.17632/bptpfpgf5h.2*

**Table 2:** Bill of materials.

Designator	Component	Qty	Cost per unit $	Total cost $	Source of materials	Material type
Heating elements	Silicone heater 100x100mm 24 V 60 W for Voron 0.1 3D printer	2	9.5	19.0	Siboor Official Store	Silicone
Cooling elements	Peltier element TEC-12706, 12 V, 60 W	2	2.7	5.4	XINCHENGXIN Store	Other
Board	Programmable controller based on ATmega328, clone of Arduino Nano V3.0	1	2.9	2.9	FAR EAST ELECTRONICS	Other
Relay	FOTEK SSR-60 DD	1	3.8	3.8	Gussy2 Store	Other
Temperature sensor	DS18B20+, 1-wire, temperature sensor	2	3.6	7.1	Intercom-NN Store	Other
Power unit for Peltier elements	Power unit Apeyron 12 V, 100 w, IP20, 8.3 A, 185x40x32 mm 03–49	1	17.6	17.6	GoodShoppingRus Store	Other
Power unit for heating elements	Power unit Apeyron 24 V, 100 w, IP20, 4.1 A, 185x40x32 mm 03–49	2	16.9	33.7	MEAN WELL 1 Store	Other
MOSFET modules	MOS Module based on FET520	2	0.6	1.1	PHINK Power Module Expert Store	Other
Heat sink for Pelteir elements	Aluminum heat sink with 1 W fan	2	2.0	4.0	SZRZGT- LED Store	Aluminum
Support for the cooling module	U-shaped 50x30x20 mm (H x D x W) mounting bracket	1	2.8	2.8	GBT-Hardware Store	Steel
Tubing	PTFE tube 0.5 mm ID 2.5 mm OD	1	2.0	2.0	Og Hardware Store	PTFE
Peristaltic pump	Any peristaltic pump, that can supply the desired flow rate	1	224.7	224.7	CHONRY Store	Other
Base plate	Any non-conducting plate (i.e. wooden or acrylic) to mount the components on.	1	3.3	3.3	DIDIHOU Official Store	Plastic or wood
Thermal paste	Any available thermal paste	1	1.1	1.1	Computercable4u	Other
Breadboard wires	Wires to assembly circuit on a breadboard	1	2	2	FYD Open Source Hardware	Other

Tubing material is of great importance. The material should be as inert and hydrophobic as possible. At high temperatures used, any protein tends to adsorb on the surface of the tubing, which is undesirable and should be avoided. Hydrophobicity is required because of the injection scheme used (see “Operation instructions“ section), as in the case of hydrophilic tubing ballast water will dilute the reaction mixture with small droplets left on the walls.

## 4 Build instructions

### 4.1 Parts preparation

1. Print the parts corresponding to 3D-models “Support for heat tables.stl” and “Pi shaped support.stl” with help of 3D-printing. Photographs of the detail are shown in Fig. 4(a, b). We used ABS plastic and 3D-printer Picasso Designer Xl Pro.

**Fig. 4:**
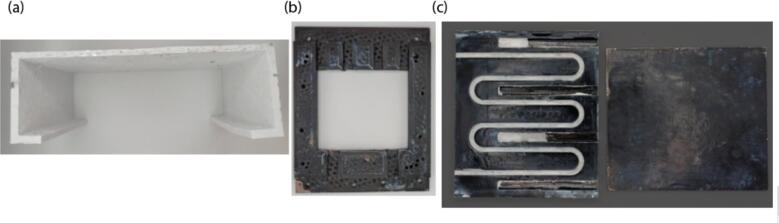
3D-printed parts and milled parts. (a) Pi-shaped support (3D-printed). (b) Support for heat tables (3D-printed). (c) flow heat cell (milled from aluminum). The upper part is shown on the right, and the bottom part (the flow cell itself) is shown on the left.
2. Mill the “flow heat cell.step” detail from aluminum or any other available metal (Fig. 4(c)).

### 4.2 Assembly of the components and circuit wiring

1. Attach the following components to the base plate via gluing or screwing: power supplies, MOSFET modules, relay, support bracket for the cooling module and a breadboard (Fig. 5(a)).

**Fig. 5:**
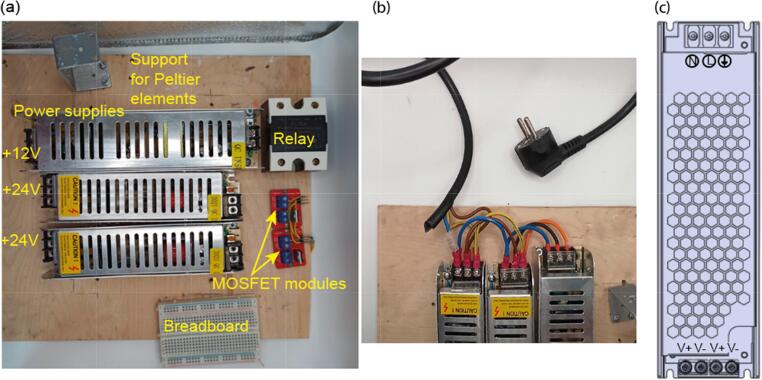
Mounting components on the base plate. (a) Location of the components on the base plate. (b) Power supply connection to an electrical plug. (c) Schematic drawing of the power supply.
2. Connect a wire with an electrical plug at one end to the input of a power supply (Fig. 5(b, c)). “L” terminal of the power supply should be connected with phase wire, which is usually of black, brown or gray color. “N” should be connected with neutral wire, which is usually blue. Finally, “GND” terminal goes to green/yellow wire. If you are not sure about the right colors, check them with a multimeter. Then, connect the input terminals with the same name of all the three power supplies. Ensure that all the connections are very secure, so they cannot accidentally slip out.
3. Wire V+ and V- terminals of 24 V power supplies to MOSFET inputs (which are also V+ and V-), carefully preserving polarity (Fig. 6(a, b)).

**Fig. 6:**
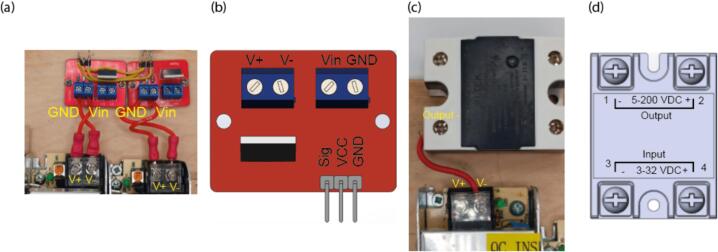
MOSFETs and relay connection. (a) Connection of the MOSFETs to the power supplies. (b) Schematic drawing of the MOSFET.(c) Connection of the relay to the power supply. (d) Schematic drawing of the relay.
4. Wire V+ terminal 24 V power supply to output “+” terminal of the relay (terminal 1), Fig. 6(c, d).
5. Plug the Arduino board on it. Connect the GND and + 5 V pin to corresponding power rails (Fig. 4b).
6. Connect the GND terminal of the MOSFET to GND power rail of the breadboard, and the VCC pin to the positive power rail. MOSFET input pins should go to pins D3, and relay input pin goes to D9 (Fig. 7).

**Fig. 7:**
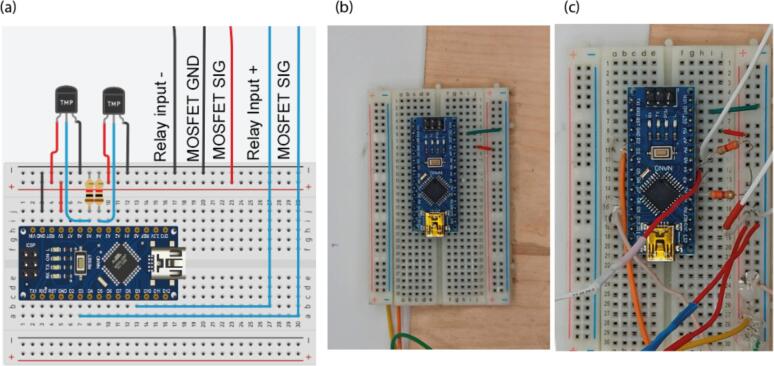
Breadboard wiring. (a) Connection of the electrical components to breadboard. (b)-(c) photographs of the breadboard before and after connection of the electrical components.
7. Connect temperature sensors. Place 4.7 kΩ resistance between A5 pin and positive power rail. Connect the GND pin of the sensor to the GND rail, + 5 V to the positive power rail, and the remaining pin to the A5 pin. Repeat the same with the second sensor and A4 pin. Make sure that for the second sensor length of wires is at least 30 cm.

### 4.3 Power electrical components

1. Place the “Pi-shaped support” for the heat tables on the base plate and glue or screw it (Fig. 8(a)). Place one of the heat tables onto it and wire it to V + and V- pins of one of the MOSFETs.

**Fig. 8:**
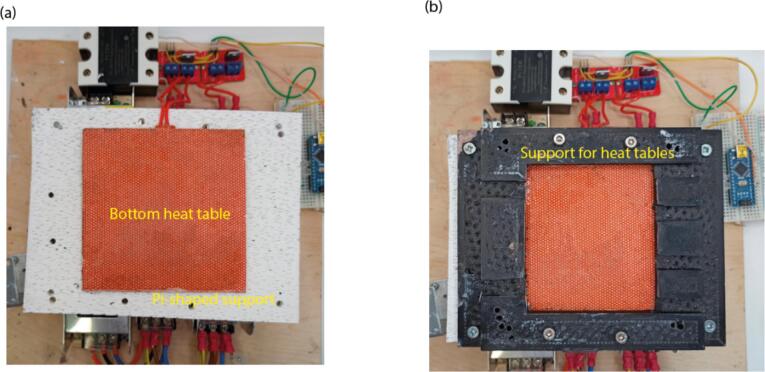
Placement of heat tables and its supports. (a) Placement of the pi shaped support and bottom heat table. (b) Placement of the support for heat tables.
2. Place the “Support for heat tables” onto the heat table and glue or screw it (Fig. 8(b)).
3. Then place parts on the support for the Peltier elements in the following order: first heat sink, first Peltier element, second Peltier element, second heat sink (Fig. 9). Apply thermal paste between the heat sink and its Peltier element.

**Fig. 9:**
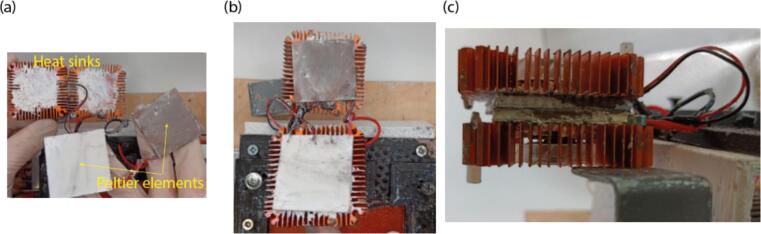
Assembly of the cooling module. (a) View of the Peltier elements and the heat sinks. (b) View of Peltier elements placed onto the heat sinks. (c) Assembled cooling module.
4. Connect the wires from heat sink fans to + 12 V power supply (Fig. 10). Then connect the Peltier elements in parallel – connect their positive and negative terminals. Then connect the positive terminal to the remaining relay output, and the negative terminal to the negative of 12 V power supply output.

**Fig. 10:**
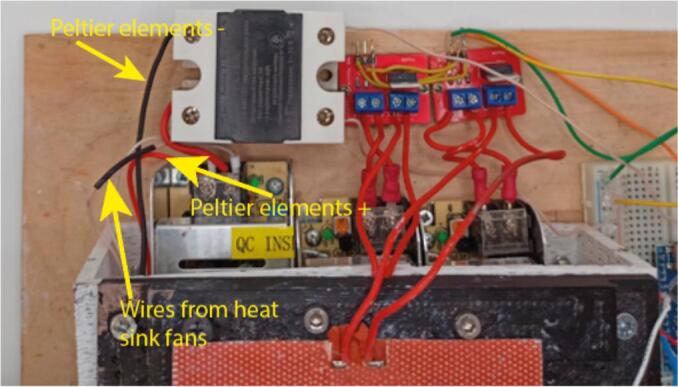
Photograph, showing wiring of the heat sinks and the Peltier elements to the relay and power supplies.
5. Place the milled flow heat cell into its support (Fig. 11 (a)). Wire the second heat table to V+ and V- pins of the second MOSFET.

**Fig. 11:**
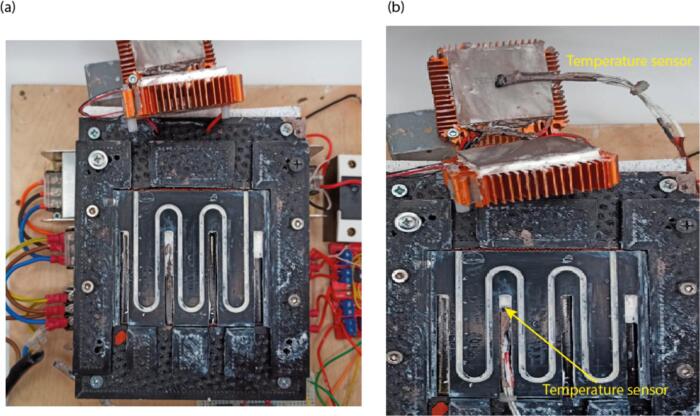
Placement of the flow cell and temperature sensors. (a) Placement of the flow cell. (b) Placement of the temperature sensors.
6. Place temperature sensors in the flow cell and between Peltier elements and apply thermal paste to the contact area (Fig. 11(b)).

### 4.4 Tubing and pump connection

1. Coat the surfaces of the flow heat cell and Peltier elements with thermal paste thoroughly (Fig. 12). Place the tubing inside the system. In case of rigid tubing (e.g., if PEEK tubing is used instead of PTFE), it can be heated with a hot air gun to bend it without kinks.

**Fig. 12:**
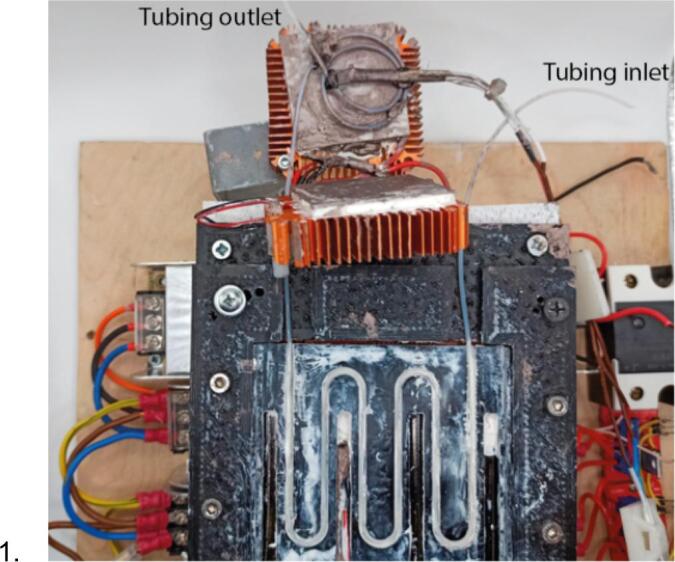
Placement of the tubing into the system.
2. Connect the peristaltic pump soft tubing (e.g., PVC, silicone) to rigid tubing of the system with, for example, soft tubing of bigger diameter (Fig. 13).

**Fig. 13:**
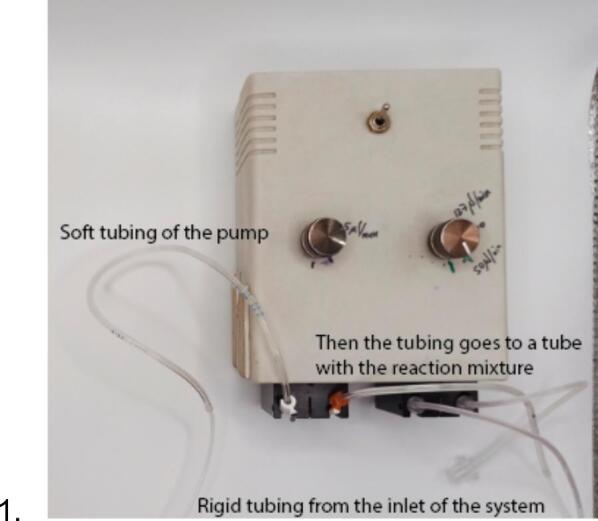
Connection of the tubing to the peristaltic pump.

After the pump connection the assembly is done. For more detailed information please refer to the 3D-model of the setup in the repository and for information on electrical components connection refer to Fig. 3.

## 5 Operation instructions

1. Connect the setup to a PC, open Arduino file, set a desired temperature for heating element in Celsius degrees, i.e., “#define SETPOINT 80”. Upload the sketch to the board.
2. Open port connection. Every second, the board sends three numbers at a time – temperature in heating element, temperature in cooling element and currently supplied power to the heating element (from 0 to 255 in a.u.)
3. Connect the device to the power supply and wait until the set temperature is reached.
4. In the meantime, prepare the synthesis mixture. To synthesize BSA nanoparticles, 10 g/l BSA solution in Milli-Q, HEPES Buffer 0.1 M pH 7.4, 20 g/l Epirubicin solution in a 1:2:1 vol ratio were mixed immediately before synthesis. Avoid using aged solutions, as Epirubicin precipitates in the pH used over the course of several hours.
5. When the temperature is reached, power the pump, and start pumping distilled water through the system at desired flow rate, to reach a stable state.
6. Turn the cooling on, by sending “n” literal to the board. Avoid prolonged operation (>20 min) with cooling module turned on, as the Peltier elements may overheat. To turn it off, send the “f” literal to the board.
7. Make sure that there is a collection tube below the outlet of the system. Inject the synthesis mixture in the system. With the pump working, lift the inlet tubing from the water reservoir and suck a bubble of approximately 50 µl into the tubing to prevent mixing of water and reaction solution. Then dip the tubing into the synthesis mixture until its depletion. Finally, suck approximately the same bubble, and dip the tubing back into the water reservoir.
8. After the synthesis turn the cooling off, and continue pumping water for several minutes. In case of tube clogging with reactants, wash the tube with concentrated alkali (i.e., 5 M NaOH). **Caution:** wear protective glasses, gloves and lab coat when performing the washing with alkali. After the alkali washing, pump the water again for another several minutes.
9. Turn the power supply off and disconnect the PC from the setup.
10. The resulting nanoparticles are centrifuged at 15,500 g for 5 min, the supernatant is removed, and the nanoparticles are resuspended in the same volume of 10 mM HEPES buffer pH 7.4.

## 6 Validation and characterization

### 6.1 Flow rate optimization

To minimize the duration of transients in the heating module and ensure strict control of the synthesis temperature, it is necessary to optimize the flow rate provided by the pump. If the flow rate is too high, the reaction mixture does not have time to reach the set temperature. On the other hand, low flow rates mean reduced throughput.

To find the maximum acceptable flow rate, the temperature distribution along the tube was studied using thermal imaging at different flow rates. The thermal imaging study was carried out with a FLIR CX7 camera. The heating module temperature was set to 90˚C. To achieve a steady state, water was continuously pumped through the system at a given flow rate for at least 5 min. After this, three IR images were taken at least 1 min apart. The resulting images were processed using ImageJ software.

**Fig. 14:**
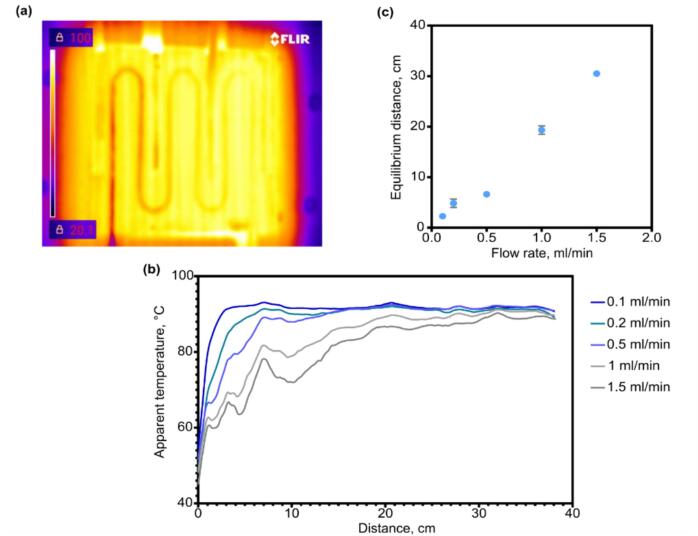
Temperature distribution study. (a) Example of IR photograph of the system. (b) Apparent temperature distribution profile at different flow rates, acquired via IR photographs analysis. (c) Distance at which reactants reach thermal equilibrium with the heating module versus the flow rate. For (b) mean values of three independent measurements are plotted. For (c) data are presented as mean ± sd.

An example of a thermogram is given in Fig. 14(a), and the temperature distribution profiles are shown in Fig. 14(b). It should be noted that the temperature depicted in Fig. 14(b) is not a real temperature of the liquid, but is rather a value in the range between the temperatures of the tubing and the reaction mixture. When a plateau is reached on the temperature distribution profile (Fig. 14(c)), the tubing and liquid come into thermal equilibrium and this point can be used to evaluate heating efficiency.

It can be seen (Fig. 14(b)) that at flow rates above 1 ml/min, the temperature almost never reaches a plateau, while at a flow rate of 0.5 ml/min, the target temperature is reached only at 20 % of the length of the heated tube. Hereinafter, we use a flow rate of 0.5 ml/min as the optimum between throughput and controllability of the synthesis.

### 6.2 Nanoparticle synthesis and scalability study

**Fig. 15:**
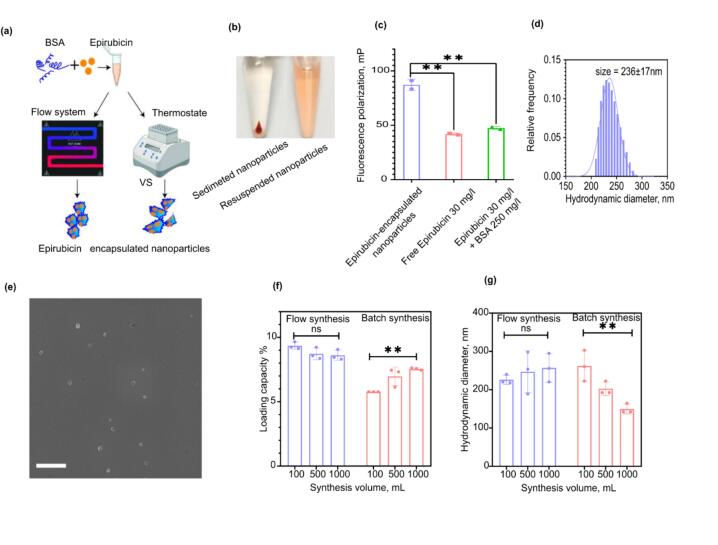
Study on nanoparticles' sizes and loading capacity. (a) Synthesis scheme. BSA and epirubicine are mixed together and are either heated in a thermostat or pumped through the flow system, resulting in nanoparticles formation. (b) Photograph of the nanoparticles, sedimented at 15500 g for 5 min (left), and resuspended after centrifugation. (c) Fluorescence polarization study of free epirubicin, free epirubicin mixed with BSA and epirubicin encapsulated nanoparticles. (d) Nanoparticles' size distribution profile (e) SEM image of the nanoparticles. The scale bar is 1 µm. (f, g). Comparison of loading capacity and hydrodynamic diameter of nanoparticles obtained via flow system and via batch synthesis in a thermostat. For (d) data are presented as mean ± distribution s.d. Each dot represents an independent synthesis.

To test the setup’s ability to produce protein nanoparticles in a scalable manner, we chose to synthesize Epirubicin-encapsulated BSA nanoparticles as a potentially clinically significant nanoagent. We also compare nanoparticles obtained using the flow system with nanoparticles obtained under similar conditions by bulk heating in a thermostat (Fig. 15(a)). To synthesize nanoparticles, a reaction mixture is prepared as described in the “Operation Instructions” section. Then it is placed in a 1.5 ml Eppendorf tube, inserted into the socket of a preheated thermoshaker TS-100 (Biosan, Latvia) to 90 °C, incubated for 1 min and immediately transferred to ice. Finally, the nanoparticles are centrifuged at 15,500 g for 5 min, the supernatant is removed and the nanoparticles are resuspended in the same volume of 10 mM HEPES buffer, pH 7.4.

The photograph of the resulting nanoparticles is shown in Fig. 15(b). The orange color of the suspension and of the pellet indicates the successful loading of nanoparticles with Epirubicin. Moreover, the nanoparticles show increased value of fluorescence polarization (Fig. 15(c)) compared to free form Epirubicin, which further confirms encapsulation. Loading capacity value (mass of drug per unit mass of the nanoparticle) comprised 8.9 ± 0.5 %. Next, nanoparticles were studied via dynamic light scattering. They have hydrodynamic diameter of 228 ± 10 nm (distribution profile is shown on Fig. 15(d)), and zeta potential of –(10 ± 2) mV at pH 7.4. The analysis was performed using Photocor Compact-Z (Photocor Ltd., Moscow, Russia) in 10 mM HEPES buffer pH 7.4, at 25 °C.

The nanoparticles demonstrate near-spherical geometry according to scanning electron microscopy (Fig. 15(e)) images, mean size is 264 ± 75 nm (mean ± distribution s.d.), which does correspond to DLS data.

Like most other BSA nanoparticles synthesis methods, STF represents a controlled protein aggregation caused by partial denaturation. Synthesis temperature and flow rate (which is in essence just synthesis duration), are the most important variables controlling the nanoparticles characteristics, such as size, yield and loading capacity (Fig. S1). Increased temperature and reduced flow rate lead to increased size of nanoparticles with concomitant drop in loading capacity. If the protein amount used is increased, a slight decrease in nanoparticle sizes is observed, with reduced protein yield (Fig. S2). Thus, changing only device-associated characteristics (flow rate and temperature) it is possible to gain control over nanoparticles sizes.

To demonstrate scalability, syntheses with overall volumes of 0.1, 0.5 and 1 ml were carried out. The change in nanoparticles’ average hydrodynamic diameter and Epirubicin loading capacity was measured (Fig. 15(f, g)). For bulk heated nanoparticles, when changing the volume from 0.1 ml to 1 ml, loading capacity changed by 30 %, and the diameter dropped by 60 %. The same parameters for nanoparticles obtained via the flow system did not change significantly (p > 0.05, two-sided Welch’s *t*-test, n = 3). Yet 1 ml was the largest synthesis scale we tested, any further increase in batch volume is not anticipated to have an impact on nanoparticles’ parameters, as the characteristic volume of the flow system is about 100 μl.

Loading capacity estimation was done as follows. BSA and Epirubicin concentrations in nanoparticles were determined via absorbance at 280 nm and 490 nm, respectively (optical density), measured using a ClarioStar microplate reader (BMG LABTECH, Ortenberg, Germany). To eliminate the influence of light scattering by the nanoparticles, 10 µl of the nanoparticles (or calibration curve solution) were mixed with 20 µl SDS 50 g/l and 70 µl of HEPES 10 mM pH 7.4. Calibration curve solutions included either Epirubicin alone in concentrations 0.5–0.00625 g/l or BSA alone in concentrations 2.5–0.01 g/l. Epirubicin concentration was determined using a calibration curve. Next, absorption of the nanoparticles at 280 nm was corrected for Epirubicin absorption, and BSA concentration was determined using a calibration curve.

### 6.3 Outlook

The system is advantageous for developing a new synthesis, as it allows to carry out optimization procedures using minimal volumes, and thus minimizing reagent consumption, which can be a limiting factor for more expensive proteins. Further, the synthesis can be easily scaled up to more significant volumes with no changes in the setup.

However, our setup has several limitations. (1) As mentioned in the Results section, the throughput is limited to 0.5 ml/min. To overcome this, a greater tube length should be placed inside the heating module, which means either an increase in the dimensions of the heating module, or denser tube packaging inside it. (2) Volumes smaller than 50 µl are difficult to work with due to the injection scheme. If smaller volumes are to be used, the diameter of the tubing should simply decrease, while the other setup parameters stay the same. (3) During optimization of a new synthesis, overly harsh conditions may lead to the formation of macroscopic aggregates, which can clog the tube. To avoid the problem, it is recommended to try out less harsh conditions (i.e. lower temperature, lower protein concentration) first, and to use chemically inert tubings (PTFE), which can be easily recovered with concentrated alkali, for example.

Compared to other millifluidic synthesis techniques [35], [36] for protein nanoparticles, STF allows for encapsulation of a small drug molecule and does not require additional polymer for the synthesis. It is also applicable for other proteins, such as antibodies [13].

Flow nature of the system, in theory, permits inline nanoparticles’ parameters control with the help of flow analyzers for online monitoring of nanoparticles properties. For example, nanoparticles size distribution may be measured with DLS, and loading capacity may be estimated with fluorescence polarization techniques (for fluorescent encapsulation agents). As a future direction, other separation and analysis techniques, such asymmetric field-flow-fractionation, size exclusion chromatography, and multi angle scattering may also be applied [38], [39].

Such integration of production and analyzing units may significantly reduce the time and resources required for optimization. Moreover, this analysis may be used together with AI techniques for tuning synthesis conditions to obtain desired physiochemical properties [40]. With the help of curiosity algorithms [41], which search for any unexpected and novel behavior, formulations with astonishing and unforeseen properties could be found.

### CRediT authorship contribution statement

**Egor S. Korenkov:** Writing – original draft, Visualization, Software, Methodology, Conceptualization. **Vladimir R. Cherkasov:** Writing – review & editing, Supervision. **Maxim P. Nikitin:** Writing – review & editing, Supervision, Conceptualization.

## Declaration of competing interest

The authors declare that they have no known competing financial interests or personal relationships that could have appeared to influence the work reported in this paper.

## Appendix A Supplementary data

Supplementary Data 1 (Final assembly.STEP)

Supplementary Data 2 (Pi shaped support.STEP)

Supplementary Data 3 (Pi shaped support.STL)

Supplementary Data 4 (Source code.ino)

Supplementary Data 5 (Support for heat table.STEP)

Supplementary Data 6 (Support for heat table.STL)

Supplementary Data 7 (Flow heat cell.STEP)

Supplementary Data 8 (Flow heat cell.STL)

Supplementary Data 9 (Circuit diagram.pdf)

Supplementary Data 10 (Supplementary.docx)

## References

[1] Dai Y., Xu C., Sun X., Chen X. (2017). Nanoparticle design strategies for enhanced anticancer therapy by exploiting the tumour microenvironment. Chem. Soc. Rev..

[2] Wahane A., Waghmode A., Kapphahn A., Dhuri K., Gupta A., Bahal R. (2020). Role of lipid-based and polymer-based non-viral vectors in nucleic acid delivery for next-generation gene therapy. Molecules.

[3] Nikitin M.P., Shipunova V.O., Deyev S.M., Nikitin P.I. (2014). Biocomputing based on particle disassembly. Nat. Nanotechnol..

[4] Kairdolf B.A., Qian X., Nie S. (2017). Bioconjugated nanoparticles for biosensing, vivo imaging, and medical diagnostics. Anal. Chem..

[5] Wolfbeis O.S. (2015). An overview of nanoparticles commonly used in fluorescent bioimaging. Chem Soc Rev.

[6] Mitchell M.J., Billingsley M.M., Haley R.M., Wechsler M.E., Peppas N.A., Langer R. (2021). Engineering precision nanoparticles for drug delivery. Nat Rev Drug Discov.

[7] Hong S., Choi D.W., Kim H.N., Park C.G., Lee W., Park H.H. (2020). Protein-Based Nanoparticles as Drug Delivery Systems. Pharmaceutics.

[8] A.S. Obozina, E.N. Komedchikova, O.A. Kolesnikova, A.M. Iureva, V.L. Kovalenko, F.A. Zavalko, T. v Rozhnikova, E.D. Tereshina, E.N. Mochalova, V.O. Shipunova, Genetically Encoded Self-Assembling Protein Nanoparticles for the Targeted Delivery In Vitro and In Vivo, Pharmaceutics 15 (2023). doi: 10.3390/pharmaceutics15010231. 10.3390/pharmaceutics15010231 PMC9861179 36678860

[9] Li H., Feng Y., Luo Q., Li Z., Li X., Gan H., Gu Z., Gong Q., Luo K. (2023). Stimuli-activatable nanomedicine meets cancer theranostics. Theranostics.

[10] Miele E., Spinelli G.P., Miele E., Tomao F., Tomao S. (2009). Albumin-bound formulation of paclitaxel (Abraxane® ABI-007) in the treatment of breast cancer. Int J Nanomed..

[11] Untch M., Jackisch C., Schneeweiss A., Conrad B., Aktas B., Denkert C., Eidtmann H., Wiebringhaus H., Kümmel S., Hilfrich J., Warm M., Paepke S., Just M., Hanusch C., Hackmann J., Blohmer J.-U., Clemens M., Darb-Esfahani S., Schmitt W.D., Dan Costa S., Gerber B., Engels K., Nekljudova V., Loibl S., von Minckwitz G. (2016). Nab-paclitaxel versus solvent-based paclitaxel in neoadjuvant chemotherapy for early breast cancer (GeparSepto—GBG 69): a randomised, phase 3 trial. LancetOncol.

[12] Tarhini M., Greige-Gerges H., Elaissari A. (2017). Protein-based nanoparticles: From preparation to encapsulation of active molecules. Int J Pharm.

[13] Lunin A.V., Korenkov E.S., Mochalova E.N., Nikitin M.P. (2021). Green Synthesis of Size-Controlled in Vivo Biocompatible Immunoglobulin-Based Nanoparticles by a Swift Thermal Formation. ACS Sustain Chem Eng.

[14] Dormont F., Rouquette M., Mahatsekake C., Gobeaux F., Peramo A., Brusini R., Calet S., Testard F., Lepetre-Mouelhi S., Desmaële D., Varna M., Couvreur P. (2019). Translation of nanomedicines from lab to industrial scale synthesis: The case of squalene-adenosine nanoparticles. J. Control. Release.

[15] Sebastian V. (2022). Toward continuous production of high-quality nanomaterials using microfluidics: nanoengineering the shape, structure and chemical composition. Nanoscale.

[16] Hakala T.A., Davies S., Toprakcioglu Z., Bernardim B., Bernardes G.J.L., Knowles T.P.J. (2020). A microfluidic co-flow route for human serum albumin-drug–nanoparticle assembly. Chem. –Eur. J..

[17] Zhang L., Beatty A., Lu L., Abdalrahman A., Makris T.M., Wang G., Wang Q. (2020). Microfluidic-assisted polymer-protein assembly to fabricate homogeneous functionalnanoparticles. Mater. Sci. Eng. C.

[18] Pourabed A., Younas T., Liu C., Shanbhag B.K., He L., Alan T. (2021). High throughput acoustic microfluidic mixer controls self-assembly of protein nanoparticles with tuneable sizes. J Colloid Interface Sci.

[19] van Ballegooie C., Man A., Andreu I., Gates B.D., Yapp D. (2019). Using a Microfluidics System to Reproducibly Synthesize Protein Nanoparticles: Factors Contributing to Size, Homogeneity, and Stability. Processes.

[20] Zhang Q., Toprakcioglu Z., Jayaram A.K., Guo G., Wang X., Knowles T.P.J. (2023). Formation of protein nanoparticles in microdroplet flow reactors. ACS Nano.

[21] Guo H., Feng Y., Deng Y., Yan T., Liang Z., Zhou Y., Zhang W., Xu E., Liu D., Wang W. (2023). Continuous flow modulates zein nanoprecipitation solvent environment to obtain colloidal particles with high curcumin loading. Food Hydrocoll.

[22] Zhao H., Wang M., Wang X., Liu J., Xing M., Huang H., Cohen Stuart M.A., Wang J. (2023). Controlled fabrication of drug-loaded protein nanoparticles via flash nanoprecipitation. AIChE J..

[23] Dressaire E., Sauret A. (2017). Clogging of microfluidic systems. Soft Matter.

[24] Li Y., Sanampudi A., Raji Reddy V., Biswas S., Nandakumar K., Yemane D., Goettert J., Kumar C.S.S.R. (2012). Size evolution of gold nanoparticles in a millifluidic reactor. ChemPhysChem.

[25] Huang H., du Toit H., Panariello L., Mazzei L., Gavriilidis A. (2021). Continuous Synthesis of Gold Nanoparticles in Micro- and Millifluidic Systems.

[26] Lohse S.E., Eller J.R., Sivapalan S.T., Plews M.R., Murphy C.J. (2013). A simple millifluidic benchtop reactor system for the high-throughput synthesis and functionalization of gold nanoparticles with different sizes and shapes. ACS Nano.

[27] Richard C., McGee R., Goenka A., Mukherjee P., Bhargava R. (2020). On-demand milifluidic synthesis of quantum dots in digital droplet reactors. Ind Eng Chem Res.

[28] Krishna K.S., Li Y., Li S., Kumar C.S.S.R. (2013). Lab-on-a-chip synthesis of inorganic nanomaterials and quantum dots for biomedical applications. Adv Drug Deliv Rev.

[29] Sitapure N., Epps R., Abolhasani M., Kwon J.-S.-I. (2021). Multiscale modeling and optimal operation of millifluidic synthesis of perovskite quantum dots: Towards size-controlled continuous manufacturing. Chem. Eng. J..

[30] Yanar F., Mosayyebi A., Nastruzzi C., Carugo D., Zhang X. (2020). Continuous-flow production of liposomes with a millireactor under varying fluidic conditions. Pharmaceutics.

[31] Yanar F., Kimpton H., Cristaldi D.A., Mosayyebi A., Carugo D., Zhang X. (2023). Synthesis and characterization of liposomes encapsulating silver nanoprisms obtained by millifluidic-based production for drug delivery. Mater. Res. Express.

[32] Engl W., Tachibana M., Panizza P., Backov R. (2007). Millifluidic as a versatile reactor to tune size and aspect ratio of large polymerized objects. Int. J. Multiph. Flow.

[33] Lukyanova L., Séon L., Aradian A., Mondain-Monval O., Leng J., Wunenburger R. (2013). Millifluidic synthesis of polymer core-shell micromechanical particles: Toward micromechanical resonators for acoustic metamaterials. J Appl Polym Sci.

[34] Garagalza O., Petit C., Mignard E., Sarrazin F., Reynaud S., Grassl B. (2017). Droplet-based millifluidic device under microwave irradiation: Temperature measurement and polymer particle synthesis. Chem. Eng. J..

[35] Chen N., Zhao J., Li S., Qi H., Li K., Li X., Hou X. (2018). Millifluidic Synthesis of Biocompatible Protein-Loaded Nanocapsules. J Nanosci Nanotechnol.

[36] Seaberg J., Kaabipour S., Hemmati S., Ramsey J.D. (2020). A rapid millifluidic synthesis of tunable polymer-protein nanoparticles. Eur. J. Pharmaceut. Biopharmaceut..

[37] Borlan R., Tatar A.-S., Soritau O., Maniu D., Marc G., Florea A., Focsan M., Astilean S. (2020). Design of fluorophore-loaded human serum albumin nanoparticles for specific targeting of NIH:OVCAR3 ovarian cancer cells. Nanotechnology.

[38] Cho T.J., Hackley V.A. (2010). Fractionation and characterization of gold nanoparticles in aqueous solution: asymmetric-flow field flow fractionation with MALS, DLS, and UV–Vis detection. Anal Bioanal Chem.

[39] Hu Y., Crist R.M., Clogston J.D. (2020). The utility of asymmetric flow field-flow fractionation for preclinical characterization of nanomedicines. Anal Bioanal Chem.

[40] Tao H., Wu T., Aldeghi M., Wu T.C., Aspuru-Guzik A., Kumacheva E. (2021). Nanoparticle synthesis assisted by machine learning. Nat. Rev. Mater..

[41] Lehman J., Stanley K.O. (2011). Abandoning objectives: evolution through the search for novelty alone. Evol Comput.

