# Supplementary figures and images for: A do-it-yourself benchtop device for highly scalable flow synthesis of protein-based nanoparticles
Source: HardwareX. 2024 Jul 1;19:e00554. doi: 10.1016/j.ohx.2024.e00554 (PMC11278078; doi:10.1016/j.ohx.2024.e00554)

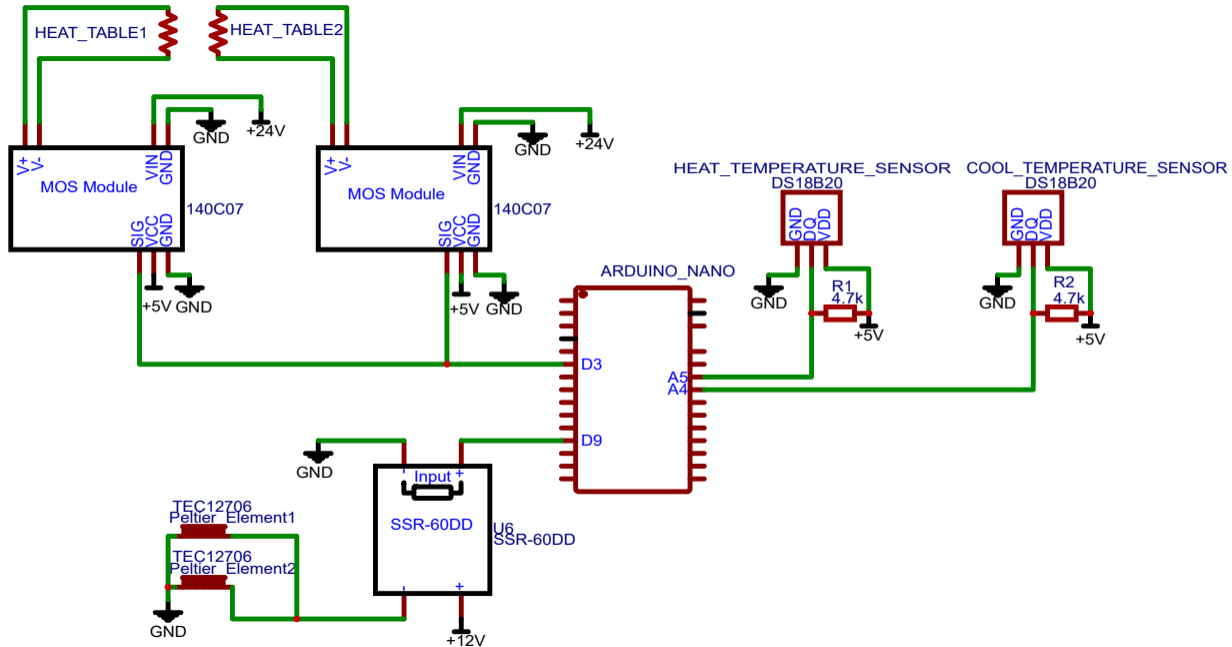

Supplement: Supplementary Data 9 — (Circuit diagram.pdf) [file mmc9.pdf]
