# Supplementary material for: A do-it-yourself benchtop device for highly scalable flow synthesis of protein-based nanoparticles
Source: HardwareX. 2024 Jul 1;19:e00554. doi: 10.1016/j.ohx.2024.e00554 (PMC11278078; doi:10.1016/j.ohx.2024.e00554)
Supplement: Supplementary Data 10 — (Supplementary.docx) [file mmc10.docx]

# Supllementary methods

## Scanning electron microscopy

Samples were prepared using HMDS drying. Briefly, the nanoparticles were additionally centrifuged (15500 g 5 min) and resuspended in a five-fold initial volume of MilliQ water.
50 µl of the nanoparticles were placed onto a silicon wafer, incubated for 10 min. Then excess (200 µl) of 96% ethanol was added on top of the drop, followed by the addition of 200 µl of HMDS. After air drying, the images were obtained with a Tescan MAIA3 microscope (Tescan, Czech Republic) at an accelerating voltage of 7 kV.

## Fluorescence polarization study

First, Epirubicin concentration was determined as described above. The fluorescence polarization was measured with 482 nm (FWHM 16 nm) excitation filter and 530 nm (FWHM 40 nm) emission filter. We compared fluorescence polarization of the nanoparticles, of the Epirubicin in the same concentration as in the nanoparticles, and of the Epirubicin in the same concentration with added BSA in concentration of 250 mg/l (the same, as used in the synthesis).

| Alternative method(s) | Brief description | Advantage of STF | Disadvantage of STF |
| --- | --- | --- | --- |
| pH coacervation, desolvation method[1–3] | Protein precipitates into nanoparticles upon organic solvent addition | Crosslinking agent is not required | Heat sensitive and water insoluble cargo cannot be used |
| Nab technology[4] | Protein solution in water and drug solution in organic solvent are mixed under high mechanical shear stress. Nanoparticles are obtained after evaporation of organic phase. | Toxic solvents are not used |  |
| Spray drying[5] | Protein solution is dried in hot air flow, forming nanoparticles | NPs of sizes less than 200 nm can be obtained |  |
| Self-assembly[6,7] | Source protein is modified to assist formation of nanoparticles | No chemical modification needed |  |
| Emulsification/Double emulsification[8] | Either w/o or w/o/w emulsion is formed, stabilized by surfactants. Nanoparticles are obtained after evaporation of organic phase. | No surfactant is used |  |

Table S1: Comparison of STF with other protein nanoparticles synthesis methods.


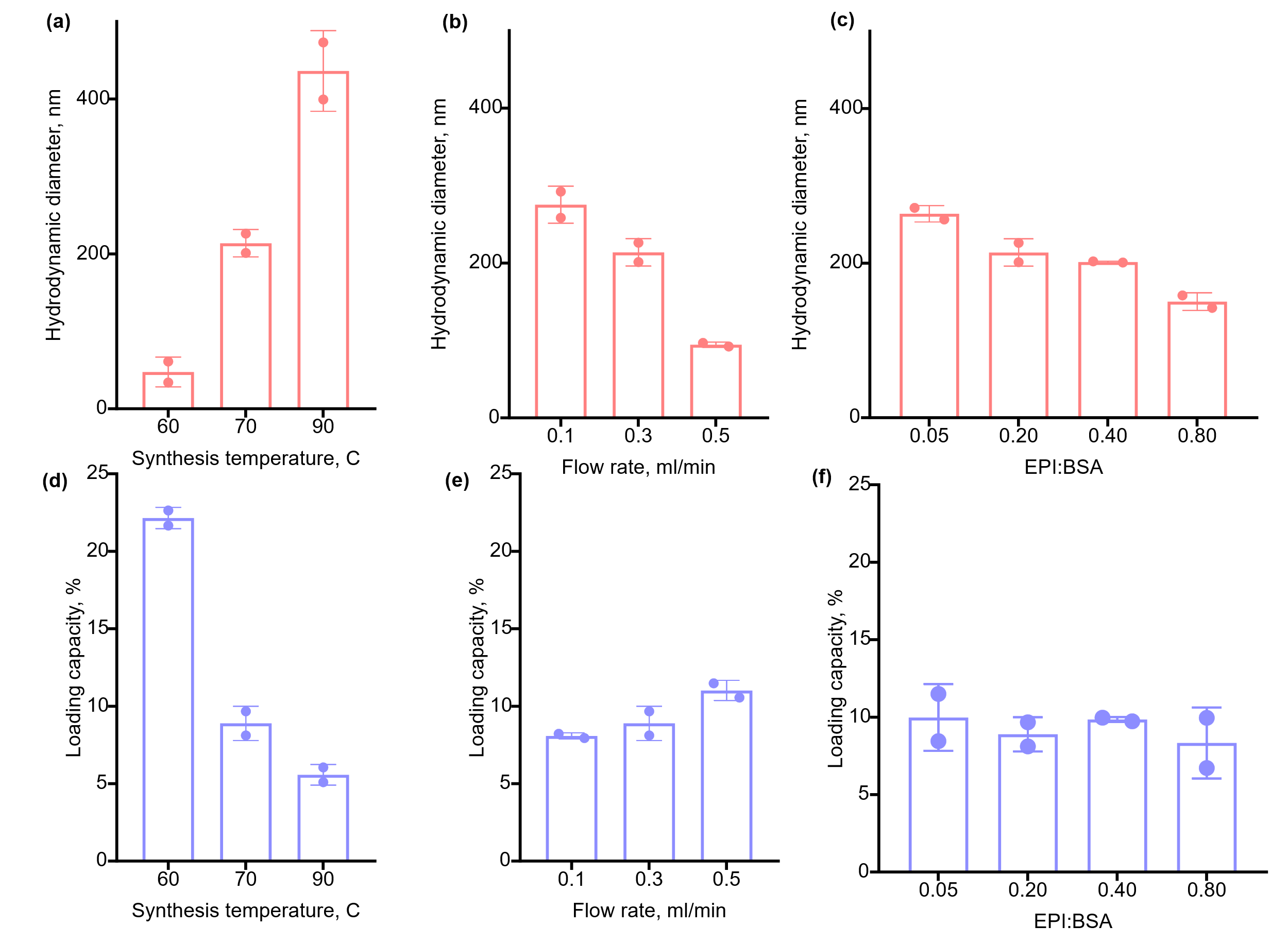


**Figure S1**. Dependence of nanoparticles size and loading capacity on synthesis parameters. (a, d) Size and loading capacity of the nanoparticles respectively versus the synthesis temperature. (b, e) Size and loading capacity of the nanoparticles respectively versus the flow rate. (c, f) Size and loading capacity of the nanoparticles respectively versus the mass ratio of Epirubicin to BSA. Unless otherwise specified, flow rate was set to 0.3 ml/min, synthesis temperature to 70 ˚C, Epirubicin and BSA concentration consituted 0.5 g/l and 2.5 g/l. Teflon tubing with ID 0.5 mm was used.


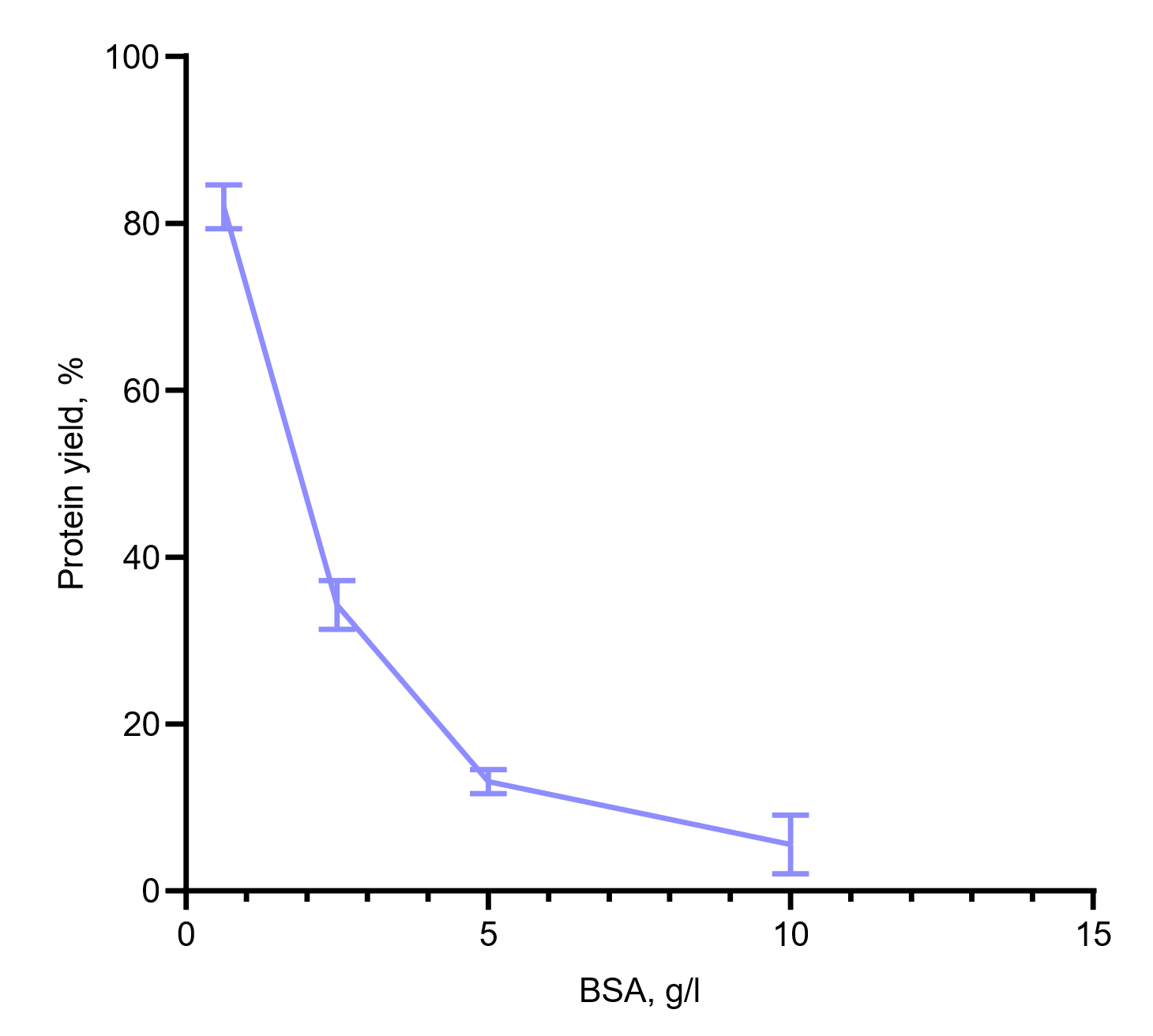


**Figure S2**. Dependence of yield versus amount of protein used.

## References

[1] A. Jahanban-Esfahlan, S. Dastmalchi, S. Davaran, A simple improved desolvation method for the rapid preparation of albumin nanoparticles, Int J Biol Macromol 91 (2016) 703–709. https://doi.org/https://doi.org/10.1016/j.ijbiomac.2016.05.032.

[2] C. Weber, C. Coester, J. Kreuter, K. Langer, Desolvation process and surface characterisation of protein nanoparticles, Int J Pharm 194 (2000) 91–102. https://doi.org/https://doi.org/10.1016/S0378-5173(99)00370-1.

[3] B. Wilson, Y. Lavanya, S.R.B. Priyadarshini, M. Ramasamy, J.L. Jenita, Albumin nanoparticles for the delivery of gabapentin: Preparation, characterization and pharmacodynamic studies, Int J Pharm 473 (2014) 73–79. https://doi.org/https://doi.org/10.1016/j.ijpharm.2014.05.056.

[4] A. Spada, J. Emami, J.A. Tuszynski, A. Lavasanifar, The Uniqueness of Albumin as a Carrier in Nanodrug Delivery, Mol Pharm 18 (2021) 1862–1894. https://doi.org/10.1021/acs.molpharmaceut.1c00046.

[5] K.Y. Wan, J. Weng, S.N. Wong, P.C.L. Kwok, S.F. Chow, A.H.L. Chow, Converting nanosuspension into inhalable and redispersible nanoparticles by combined in-situ thermal gelation and spray drying, European Journal of Pharmaceutics and Biopharmaceutics 149 (2020) 238–247. https://doi.org/https://doi.org/10.1016/j.ejpb.2020.02.010.

[6] A.S. Obozina, E.N. Komedchikova, O.A. Kolesnikova, A.M. Iureva, V.L. Kovalenko, F.A. Zavalko, T. v Rozhnikova, E.D. Tereshina, E.N. Mochalova, V.O. Shipunova, Genetically Encoded Self-Assembling Protein Nanoparticles for the Targeted Delivery In Vitro and In Vivo, Pharmaceutics 15 (2023). https://doi.org/10.3390/pharmaceutics15010231.

[7] T. Lin, P. Zhao, Y. Jiang, Y. Tang, H. Jin, Z. Pan, H. He, V.C. Yang, Y. Huang, Blood–Brain-Barrier-Penetrating Albumin Nanoparticles for Biomimetic Drug Delivery via Albumin-Binding Protein Pathways for Antiglioma Therapy, ACS Nano 10 (2016) 9999–10012. https://doi.org/10.1021/acsnano.6b04268.

[8] S. Ding, C.A. Serra, T.F. Vandamme, W. Yu, N. Anton, Double emulsions prepared by two–step emulsification: History, state-of-the-art and perspective, Journal of Controlled Release 295 (2019) 31–49. https://doi.org/https://doi.org/10.1016/j.jconrel.2018.12.037.
